# Risk factors for in-hospital mortality after total arch procedure in patients with acute type A aortic dissection

**DOI:** 10.3389/fcvm.2023.1149907

**Published:** 2023-04-25

**Authors:** Zhao An, Keng Zhong, Yangyong Sun, Lin Han, Zhiyun Xu, Bailing Li

**Affiliations:** ^1^ Department of Thoracic Surgery, Shanghai Pulmonary Hospital, Tongji University School of Medicine, Shanghai, China; ^2^ Department of Cardiovascular Surgery, Changhai Hospital, Naval Medical University, Shanghai, China; ^3^ Department of Cardiothoracic Surgery, Affiliated People's Hospital of Jiangsu University, Zhenjiang, China

**Keywords:** acute type A aortic dissection, in-hospital mortality, total arch procedure, risk factors, surgical repair

## Abstract

**Object:**

Knowledge about the risk factors of in-hospital mortality for acute type A aortic dissection (ATAAD) patients who received total arch procedure is limited. This study aims to investigate preoperative and intraoperative risk factors of in-hospital mortality of these patients.

**Methods:**

From May 2014 to June 2018, 372 ATAAD patients received the total arch procedure in our institution. These patients were divided into survival and death groups, and patients` in-hospital data were retrospectively collected. Receiver operating characteristic curve analysis was adopted to determine the optimal cut-off value of continuous variables. Univariate and multivariable logistic regression analyses were used to detect independent risk factors for in-hospital mortality.

**Results:**

A total of 321 patients were included in the survival group and 51 in the death group. Preoperative details showed that patients in the death group were older (55.4 ± 11.7 vs. 49.3 ± 12.6, *P* = 0.001), had more renal dysfunction (29.4% vs. 10.9%, *P* = 0.001) and coronary ostia dissection (29.4% vs. 12.2%, *P* = 0.001), and decreased left ventricular ejection fraction (LVEF) (57.5 ± 7.9% vs. 59.8 ± 7.3%, *P* = 0.032). Intraoperative results showed that more patients in the death group experienced concomitant coronary artery bypass grafting (35.3% vs. 15.3%, *P* = 0.001) with increased cardiopulmonary bypass (CPB) time (165.7 ± 39.0 vs. 149.4 ± 35.8 min, *P* = 0.003), cross-clamp time (98.4 ± 24.5 vs. 90.2 ± 26.9 min, *P* = 0.044), and red blood cell transfusion (913.7 ± 629.0 vs. 709.7 ± 686.6 ml, *P* = 0.047). Logistic regression analysis showed that age >55 years, renal dysfunction, CPB time >144 min, and RBC transfusion >1,300 ml were independent risk factors for in-hospital mortality in patients with ATAAD.

**Conclusion:**

In the present study, we identified that older age, preoperative renal dysfunction, long CPB time, and intraoperative massive transfusion were risk factors for in-hospital mortality in ATAAD patients with the total arch procedure.

## Introduction

Acute type A aortic dissection (ATAAD) is a lethal medical emergency, and surgical intervention is inevitable. The surgical strategy of ATAAD has experienced continuous evolution and our previous study showed that total arch replacement with the frozen elephant trunk (FET) technique had advantage compared with hemiarch replacement (1). Although studies showed that total arch replacement with FET technique was an effective way for the treatment of ATAAD, the in-hospital mortality of ATAAD still ranged from 4.7% to 25.7%, which was relatively high (1–5).

Studies have showed that some factors were associated with postoperative prognosis in ATAAD patients. It was reported that preoperative acute kidney injury, cardiovascular disease history, aortic root procedure, and postoperative 24 h drainage were prognostic risk factors for patients with ATAAD (6–8). However, these studies included different surgical strategies in arch repair, and knowledge in the risk factors for ATAAD patients who received the total arch procedure was very limited.

In the present study, we retrospectively collected data of patients with ATAAD who received the total arch procedure in our institution and tried to find the preoperative and intraoperative risk factors of in-hospital mortality.

## Methods

### Patients

Data of ATAAD patients who received the total arch procedure in our institution between May 2014 and June 2018 were retrospectively collected, which included the general information, perioperative details, and complications. In this study, renal dysfunction was defined as estimated glomerular filtration rate (eGFR) <60 ml/min. A total of 372 patients were included in this study, 321 patients were in the survival group [243 (75.7%) were male] and 51 patients [36 (70.6%) were male] were in the death group. This study was approved by the Institutional Review Board of Changhai hospital, and informed consent was acquired from the patients or their direct relatives before operation.

### Surgical procedure

Total arch replacement with the FET technique was adopted in this study. In brief, all surgical procedures were performed under general anesthesia, median sternotomy, and deep hypothermia. Cardiopulmonary bypass (CPB) was established with cannulation in the left or right femoral artery, right axillary artery, superior, and inferior vena cava. Ascending aorta was incised and explored after cross-clamp was placed, and the root procedure was performed during cooling. Once the rectal temperature reached 25°C–27°C, CPB was arrested and selective cerebral perfusion (SCP) was started. Then, the cross-clamp in the aorta was removed, and we explored the arch, descending aorta, and brachiocephalic arteries to determine the intimal tear and dissection range. A four-branched vascular prosthesis (Boston Scientific Inc., Boston, MA, United States) was adopted for the proximal anastomose with an FET (MicroPort Medical Co Ltd, Shanghai, China) in the descending aortic true lumen. The FET was anastomosed to the distal end of the four-branched vascular prosthesis with the descending aortic wall. After air exhaust, CPB was restarted with one branch and brachiocephalic arteries were anastomosed to the resident three branches during rewarming.

### Statistical analysis

For continuous variables, Student’s t test was adopted for statistical analysis. For categorical variables, chi-square test was used for statistical analysis. Receiver operating characteristic (ROC) curve analysis was used to get the optimal cut-off value. Univariate and multivariate logistic regression analyses were used to find the independent risk factors for in-hospital mortality. Odds ratio (OR) and 95% confidence interval (CI) were adopted to measure the risk factors` effect on mortality. SPSS (Chicago, IL, United States) was used for statistical analysis. Data were presented as mean ± SD or *n* (%). *P* < 0.05 was considered for statistical significance.

## Results

### Patient characteristics

Preoperative patients` details showed that patients in death group were older (55.4 ± 11.7 vs. 49.3 ± 12.6 years, *P *= 0.001), with more renal dysfunction (29.4% vs. 10.9%, *P *= 0.001), coronary ostia dissection (29.4% vs. 12.2%, *P *= 0.001), and decreased left ventricular ejection fraction (LVEF) (57.5 ± 7.9 vs. 59.8 ± 7.3, *P *= 0.032). Details of this part are shown in Table 1.

**Table 1:** Patient characteristics.

	Survival (*N* = 321)	Death (*N* = 51)	*P*
Age (years)	49.3 ± 12.6	55.4 ± 11.7	0.001
Gender (male)	243 (75.7)	36 (70.6)	0.43
BMI	26.3 ± 14.5	24.5 ± 3.9	0.38
Marfan	30 (9.4)	2 (3.9)	0.20
BAV	6 (1.9)	1 (2.0)	1.00
Renal dysfunction*	35 (10.9)	15 (29.4)	0.001
Smoker	97 (30.2)	18 (35.3)	0.47
Hypertension	190 (59.2)	36 (70.6)	0.12
Diabetes	14 (4.4)	2 (3.9)	1.00
Stroke history	11 (3.4)	1 (2.0)	0.90
LVEF (%)	59.8 ± 7.3	57.5 ± 7.9	0.032
MD of ascending aorta (cm)	4.6 ± 1.0	4.6 ± 0.9	0.96
Coronary ostia dissection	39 (12.2)	15 (29.4)	0.001
Aortic regurgitation ≥ Moderate	111 (34.6)	20 (39.2)	0.52
Pericardial tamponade	75 (23.4)	17 (33.3)	0.13

BMI, body mass index; BAV, bicuspid aortic valve; LVEF, left ventricular ejection fraction; MD, maximal diameter; eGFR, estimated glomerular filtration rate.

Data presented as mean ± SD or *n* (%).

Red colour values in this Table indicate statistical significance; *P* < 0.05.

*eGFR < 60 ml/min.

### Surgical procedure

Patients` intraoperative details are shown in Table 2. Results showed that more patients had concomitant coronary artery bypass grafting (CABG) in the death group (35.3% vs. 15.3%, *P *= 0.001). CPB and cross-clamp time was longer in the death group (165.7 ± 39.0 vs. 149.4 ± 35.8, *P *= 0.003; 98.4 ± 24.5 vs. 90.2 ± 26.9 min, *P *= 0.044). Patients in the death group had more red blood cells (RBCs) transfusion (913.7 ± 629.0 vs. 709.7 ± 686.6 ml, *P *= 0.047).

**Table 2:** Intraoperative results.

	Survival (*N* = 321)	Death (*N* = 51)	*P*
Rupture area			
Ascending aorta	158 (49.2)	25 (49.0)	0.98
Arch	142 (44.2)	21 (41.2)	0.68
Descending aorta	21 (6.5)	5 (9.8)	0.58
Root procedure			
Bentall	109 (34.0)	16 (31.4)	0.72
David	13 (4.1)	1 (2.0)	0.74
Root reconstruction	199 (62.0)	34 (66.7)	0.52
Concomitant CABG	49 (15.3)	18 (35.3)	0.001
CPB time (min)	149.4 ± 35.8	165.7 ± 39.0	0.003
SCP time (min)	26.1 ± 11.1	29.0 ± 20.2	0.13
Cross-clamp time (min)	90.2 ± 26.9	98.4 ± 24.5	0.044
RBCs (ml)	709.7 ± 686.6	913.7 ± 629.0	0.047
PLTs (unit)	10.6 ± 4.1	11.4 ± 4.5	0.20

CABG, coronary artery bypass grafting; CPB, cardiopulmonary bypass; SCP, selective cerebral perfusion; RBCs, red blood cells; PLTs, platelets.

Red colour values in this Table indicate statistical significance; *P* < 0.05.

Data presented as mean ± standard deviation or *n* (%).

### Outcomes

In-hospital mortality was 13.7%. Postoperative data comparison between death and survival groups showed more postoperative 24 h chest drainage in the death group (803.1 ± 583.5 vs. 537.3 ± 325.0 ml, *P *= 0.002). More patients experienced reoperation for bleeding, ventilation time ≥72 h, stroke, and acute renal failure (ARF) needing hemodialysis in the death group (19.6% vs. 2.8%, *P *= 0.001; 74.5% vs. 22.7%, *P *= 0.001; 13.7% vs. 1.9%, *P* = 0.001; 76.5% vs. 6.5%, *P* = 0.001). Details of the postoperative results are shown in Table 3.

**Table 3:** Postoperative results.

	Survival (*N* = 321)	Death (*N* = 51)	*P*
ICU time (days)	9.1 ± 10.2	12.8 ± 15.8	0.11
24 h chest drainage (ml)	537.3 ± 325.0	803.1 ± 583.5	0.002
Reoperation for bleeding	9 (2.8)	10 (19.6)	0.001
Ventilation time ≥72 h	73 (22.7)	38 (74.5)	0.001
Stroke	6 (1.9)	7 (13.7)	0.001
ARF need hemodialysis*	21 (6.5)	39 (76.5)	0.001
Paraplegia	3 (0.9)	1 (2.0)	1.00
Paraparesis	9 (2.8)	2 (3.9)	1.00
Hydropericardium need drainage	43 (13.4)	4 (7.8)	0.51

ICU, intensive care unit; ARF, acute renal failure; eGFR, estimated glomerular filtration rate.

Data presented as mean ± SD or *n* (%).

Red colour values in this Table indicate statistical significance; *P* < 0.05.

*eGFR < 60 ml/min and need hemodialysis.

### Receiver operating characteristic curve analysis

**Figure 1:**
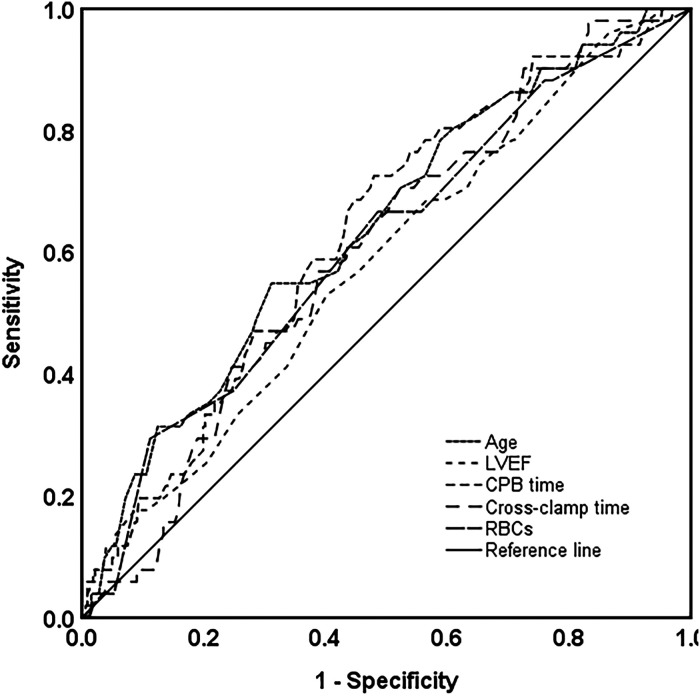
Survival prediction with ROC analysis based on age, LVEF, CPB, and cross-clamp time and intraoperative RBC transfusion. ROC, receiver operating characteristic; LVEF, left ventricular ejection fraction; CPB, cardiopulmonary bypass; RBCs, red blood cells.

For continuous variables that had statistical differences between the death and survival groups, ROC curve analysis was adopted to find the optimal cut-off values. With the ROC curve analysis, optimal cut-off value of age, CPB time, cross-clamp time, and RBC transfusion were 55 years, 144 min, 84 min, and 1,300 ml, respectively. Details of the ROC analysis are shown in Figure 1 and Table 4.

**Table 4:** Details of the ROC curve analysis.

	Area under the curve	*P*	Cut-off value
Age	0.64	0.002	55
LVEF	0.58	0.06	—
CPB time	0.63	0.003	144
Cross-clamp time	0.60	0.02	84
RBCs (ml)	0.61	0.012	1,300

ROC, receiver operating characteristic; LVEF, left ventricular ejection fraction; CPB, cardiopulmonary bypass; RBCs, red blood cells.

Red colour values in this Table indicate statistical significance; *P* < 0.05.

### Risk factors of in-hospital mortality

We used univariate and multivariable logistic regression analyses to detect in-hospital death risk factors for patients with ATAAD (Table 5). Univariate logistic regression analyses results showed that age >55 years, preoperative renal dysfunction, coronary ostia dissection, concomitant CABG, prolonged CPB and cross-clamp time, and more intraoperative RBC transfusion were the risk factors for postoperative mortality. Then, we further analyzed these seven risk factors with multivariable logistic regression analysis, and the results showed that age >55 years, preoperative renal dysfunction, CPB time >144 min, and intraoperative RBC transfusion >1,300 ml were four independent risk factors for in-hospital mortality in patients with ATAAD who received total arch procedure.

**Table 5:** Univariate and multivariable logistic regression analysis for in-hospital death risk factors in patients with ATAAD.

Variables	Univariate		*P*	Multivariate		*P*
	OR	95% CI		OR	95% CI	
Age >55 years	2.69	1.48–4.90	0.001	3.14	1.61–6.12	0.001
Renal dysfunction	3.41	1.70–6.84	0.001	3.56	1.65–7.70	0.001
Coronary ostia dissection	3.01	1.51–6.00	0.002	1.44	0.59–3.50	0.42
Concomitant CABG	3.03	1.58–5.80	0.001	2.24	0.94–5.34	0.07
CPB time >144 min	2.90	1.51–5.58	0.001	2.30	1.01–5.24	0.049
Cross-clamp time >84 min	2.19	1.15–4.15	0.017	1.36	0.59–3.13	0.47
RBCs >1,300 ml	3.30	1.65–6.61	0.001	2.70	1.25–5.84	0.012

ATAAD, acute type A aortic dissection; CABG, coronary artery bypass grafting; CPB, cardiopulmonary bypass; RBCs, red blood cells; OR, odds ratio; CI, confidence interval.

Red colour values in this Table indicate statistical significance; *P* < 0.05.

## Discussion

Surgical treatment of ATAAD is still a challenge with high in-hospital mortality (9). However, in-hospital mortality risk factors of ATAAD have not been fully clarified because of the limited number of patients and few studies systematically focused on this field. We identified four independent risk factors for in-hospital mortality of ATAAD in the present study.

Previous studies showed that surgical mortality of ATAAD increased with age. Trimarchi et al. reported that age ≥70 years was an independent predictor of mortality, but the mortality of surgical treatment was still lower compared with medical treatment (10). Another study also found that in-hospital mortality in ATAAD patients aged ≥70 years was increased (11). However, there was also a study that showed age >80 years was not associated with increased in-hospital mortality (12). However, patients received total arch procedure in these studies were limited, and we found that age >55 years was an independent risk factor of mortality in ATAAD patients who received total arch procedure.

Fan et al. found that preoperative renal dysfunction was a risk factor of in-hospital mortality in ATAAD patients; they also found that patients with increased preoperative serum creatinine had lower 90-day survival rates (13). Imasaka et al. found that preoperative renal dysfunction predicted the need for postoperative renal replacement therapy (14), which was an important risk factor for in-hospital death in ATAAD patients (15). In this study, we found that preoperative renal dysfunction was an independent risk factor for in-hospital mortality in ATAAD patients.

We reviewed studies on CPB time in ATAAD patients and found that it was a risk factor for in-hospital mortality (16). Zhang et al. found that the duration of CPB time in ATAAD surgery was associated with postoperative increased mortality (17). That study also showed that CPB time was an independent predictor of postoperative acute renal dysfunction in ATAAD patients (18). These findings were in accordance with our study, and we found that prolonged CPB time was a promoting factor for the in-hospital mortality.

Intraoperative blood transfusion volume in ATAAD surgery is associated with postoperative adverse events. Liu et al. found that intraoperative blood transfusion was an independent risk factor for postoperative renal dysfunction (19). Naeem et al. found that intraoperative blood transfusion predicted prolonged postoperative length of stay and infection; they also found that patients with increased intraoperative blood transfusion had increased mortality, although there was no statistical difference (20). We found that increased intraoperative blood transfusion was an independent predictor for postoperative mortality in ATAAD patients.

It was reported that postoperative prolonged ventilation time occurred in about 28.9% ATAAD patients and it was associated with an increased in-hospital mortality (21). In the present study, we found that 74.5% patients in the death group had ventilation time more than 72 h, which was significantly higher than the survival group. It was reported that postoperative stroke occurred in about 15.8% ATAAD patients and data from the German Registry for Acute Aortic Dissection Type A (GERAADA) showed it was related with an increased postoperative mortality (22, 23). Our study also showed that stroke in the death group was significantly higher than the survival group. We found that 76.5% patients got postoperative ARF need hemodialysis in the death group. Sansone et al. found that postoperative 30-day mortality in ATAAD patients with ARF was 50%, which was 13% much higher than in patients with no ARF (24). These studies were consistent with our findings and indicated that ATAAD patients with postoperative prolonged ventilation time, stroke, and ARF had poor prognosis. However, in this study, we focused on the preoperative and intraoperative risk factors for in-hospital mortality and we did not include postoperative factors in the logistic regression analysis.

Our study has some limitations. First, this study was an in-hospital short-term study and the effect of these predictors on the long-term prognosis of ATAAD was unclear. Second, our study was based on a single-center experience, and a multicenter study is needed to further verify findings in this study.

## Conclusion

In the present study, we identified that older age, preoperative renal dysfunction, long CPB time, and intraoperative massive transfusion were the risk factors for in-hospital mortality in ATAAD patients with the total arch procedure.

## Data Availability

The original contributions presented in the study are included in the article, further inquiries can be directed to the corresponding authors.
